# Statistical Mechanics of Non-Muscle Myosin IIA in Human Bone Marrow-Derived Mesenchymal Stromal Cells Seeded in a Collagen Scaffold: A Thermodynamic Near-Equilibrium Linear System Modified by the Tripeptide Arg-Gly-Asp (RGD)

**DOI:** 10.3390/cells9061510

**Published:** 2020-06-21

**Authors:** Yves Lecarpentier, Vincent Kindler, Xénophon Krokidis, Marie-Luce Bochaton-Piallat, Victor Claes, Jean-Louis Hébert, Alexandre Vallée, Olivier Schussler

**Affiliations:** 1 Centre de Recherche Clinique, Grand Hôpital de l’Est Francilien, 77104 Meaux, France; xkrokidis@gmail.com; 2 Department of Specialties in Medicine, Hematology Service, Geneva University Hospital, Faculty of Medicine, 1200 Geneva, Switzerland; Vincent.Kindler@unige.ch; 3 Department of Pathology and Immunology, Geneva University Hospital, Faculty of Medicine, 1200 Geneva, Switzerland; Marie-Luce.Piallat@unige.ch; 4 Department of Pharmaceutical Sciences, University of Antwerp, 2000 Wilrijk, Belgium; victor.claes@scarlet.be; 5 Institut de Cardiologie, Hôpital de la Pitié-Salpêtrière, 75013 Paris, France; jean.l.hebert@gmail.com; 6 Diagnosis and Therapeutic Center, Hypertension and Cardiovascular Prevention Unit, Hôtel-Dieu Hospital, AP-HP, Paris-Descartes University, 75004 Paris, France; alexandre.g.vallee@gmail.com; 7 Department of Cardiovascular Surgery, Research Laboratory, Geneva University Hospital, Faculty of Medicine, 1200 Geneva, Switzerland; olivier.schussler@gmail.com; 8 Department of Thoracic surgery, Nouvel Hôpital Civil, Hôpitaux Universitaires de Strasbourg, 67000 Strasbourg, France

**Keywords:** myofibroblast, mesenchymal stromal cell, RGD, collagen scaffold, tissue engineering, bone marrow, non-muscle myosin NMMIIA, statistical mechanics, entropy production rate, chemical affinity, thermodynamic force, thermodynamic flow, near-equilibrium thermodynamics, linear stationary state

## Abstract

Mesenchymal stromal cells (MSCs) were obtained from human bone marrow and amplified in cultures supplemented with human platelet lysate. Once semi-confluent, cells were seeded in solid collagen scaffolds that were rapidly colonized by the cells generating a 3D cell scaffold. Here, they acquired a myofibroblast phenotype and when exposed to appropriate chemical stimulus, developed tension and cell shortening, similar to those of striated and smooth muscle cells. Myofibroblasts contained a molecular motor—the non-muscle myosin type IIA (NMMIIA) whose crossbridge (CB) kinetics are dramatically slow compared with striated and smooth muscle myosins. Huxley’s equations were used to determine the molecular mechanical properties of NMMIIA. Thank to the great number of NMMIIA molecules, we determined the statistical mechanics (SM) of MSCs, using the grand canonical ensemble which made it possible to calculate various thermodynamic entities such as the chemical affinity, statistical entropy, internal energy, thermodynamic flow, thermodynamic force, and entropy production rate. The linear relationship observed between the thermodynamic force and the thermodynamic flow allowed to establish that MSC-laden in collagen scaffolds were in a near-equilibrium stationary state (affinity ≪ RT), MSCs were also seeded in solid collagen scaffolds functionalized with the tripeptide Arg-Gly-Asp (RGD). This induced major changes in NMMIIA SM particularly by increasing the rate of entropy production. In conclusion, collagen scaffolds laden with MSCs can be viewed as a non-muscle contractile bioengineered tissue operating in a near-equilibrium linear regime, whose SM could be substantially modified by the RGD peptide.

## 1. Introduction

Several “non-muscle” native tissues, including the placenta and the skin or pathological or bioengineered tissues can develop shortening and/or tension under specific circumstances when they are stimulated by an electrical field or KCl in vitro. Most of these “non-muscle” structures can be native, bioengineered or pathological tissues. A major common characteristic of these contractile tissues is that they contain numerous myofibroblasts, that are known to be the effector cells of tissue contraction. Myofibroblasts have been discovered in the skin by Gabbiani et al. [1]. These cells are involved in wound healing contraction secondary to their active retraction in the granulation tissue [2]. Myofibroblasts are also abundant in normal non-inflammatory tissues such as human placenta [3] and account for its contractile properties [4,5,6]. Numerous myofibroblasts are present in pathological tissues, such as cancer stroma, and in fibrotic processes [7,8,9]. Finally, considering bioengineered tissues, human medullar MSCs seeded into collagen scaffolds display contractile features, similar to those of the human placenta [10]. Myofibroblast contractility is empowered by the molecular motor NMMIIA [11,12]. The main characteristic of this molecular motor is the dramatically low kinetics of its actin-myosin crossbridges (CBs) [13,14]. 

The extensive number of myosin molecules present in contractile tissues provides the prerequisite required for using statistical mechanics (SM) [15] to describe the molecular process. Such a protocol allows the study of the thermodynamic properties of muscle and non-muscle contractile systems [16]. The cornerstone of this methodology is provided by the A. Huxley formalism [17] which allows to determine the probabilities of each of the different states of the myosin CB during the cell contractile cycle [18,19]. Thus SM, combined with A. Huxley’s formalism [17], allows to calculate the molecular partition function of the system under study [20]. Moreover several macroscopic properties of the system such as statistical entropy, chemical affinity and internal energy that are byproducts of the molecular partition function can also be determined [15]. The grand canonical ensemble is a general method if one wants to use SM for studying complex open systems. Living organisms such as muscle or non-muscle tissues are open systems which do not behave in thermodynamic equilibrium but function near thermodynamic equilibrium [16,21]. These living systems can be maintained in a near-equilibrium state thanks to a carefully balanced flow of energy and matter. Under specific conditions, a system standing near equilibrium can remain in a stationary state, whereby the linear phenomenological laws of Onsager are valid, (i.e., where the thermodynamic force varies linearly with the thermodynamic flow) [21,22]. The linear near-equilibrium thermodynamics has been applied to several biological systems [23,24,25,26]. In the present study, we used the grand canonical ensemble to human bone marrow-derived MSCs seeded in a collagen scaffold in order to determine their thermodynamic properties. We showed that the bioengineered tissue we generated exhibited a near-equilibrium thermodynamic status associated with a linear stationary state. Furthermore, statistical mechanics of native collagen scaffolds laden with MSCs was substantially modified when collagen was functionalized with the tripeptide Arg-Gly-Asp (RGD).

## 2. Materials and Methods

The local ethics committee of Geneva University Hospital, the “Commission Cantonale Ethique de la Recherche Scientifique de Genève” (CCER) approved the study (“Etude des cellules stromales issues de la moelle non-hématopoïetique des têtes et des condyles de femurs”; reference number: CER: 01-172; CHIR 01-015). The date of the Ethical Approval was 20 June 2006. Human femoral heads were collected during surgical interventions for hip replacements, in agreement with the CCER. Patients were informed and gave their written consent. MSC extraction, amplification, seeding in 3D-solid collagen scaffolds and basic contraction characteristics have been previously described [27,28]. The present experiments were performed with the same type of MSC-laden collagen scaffolds used in a previous study [10]. Scaffolds consisted in clinical hemostatic sponges (Avitene™ ultrafoam collagen, ref. 1050050, Bard Ltd., Crawley, UK,) and were solid, highly, porous collagen scaffold obtained by physical reticulation by dehydrothermal (DHT) of bovin dermal type I and type III collagens. The phenotype of myofibroblasts was determined by staining membrane cross sections with anti-*α*SMA, anti-SMMHCs, and anti-NMMHCIIA antibodies. This showed that cells constituting the surface layer and those residing in the inner space of the membrane expressed *α*SMA and NMMHCIIA, but not SMMHCs. Moreover, confocal microscopy analysis demonstrated that *α*SMA and NMMHCIIA colocalized within the same stress fibers in MSCs grown in collagen scaffolds. Characterization of the myofibroblast phenotype was also confirmed by the contractile properties and the ultraslow kinetics of the NMMIIA molecular motors. The functionalization of the solid clinical hemostatic sponge by the covalent binding with the linear RGD motive of fibronectin i.e., glycine-arginine-glycine-aspartic acid-serine peptide (GRGDS; G4391, Sigma Aldrich, Lyon, France) has been reported in an earlier study [27].

### 2.1. Experimental Set-Up

MSC-seeded scaffold was mounted in a tissue chamber containing a Krebs-Henseleit solution (in mM): 118 NaCl; 24 NaHCO_3_; 4.7 KCl; 1.2 MgSO_4_ (7H_2_O); 1.1 KH_2_PO_4_; 2.5 CaCl_2_ (6H_2_O); 4.5 glucose. The solution was bubbled with 95% O_2_-5% CO_2_ to maintain a pH at 7.4. Contractile scaffolds were chemically stimulated by means of K Cl (0.05 M). The resting tension (RT) was the load imposed on the scaffold at rest and which induced neither shortening nor lengthening. At the end of the experiment, the scaffold cross-sectional area (CSA) (in mm^2^) was calculated from the ratio of the scaffold weight and the scaffold length (Lo). The experimental procedure and the electromagnetic lever system have been described in an earlier study [6]. The resting length (Lo) was the length at RT and was equal to 5.0 ± 1.0 mm in COL-MSC scaffolds (*n* = 14 from 14 donors) and 4.8 ± 1.1 mm in COL-MSC-RGD scaffolds (*n* = 10 from 10 donors). RT was equal to 0.08 ± 0.07 mN/mm^2^ in COL-MSC scaffolds and 0.10 ± 0.08 mN/mm^2^ in COL-MSC-RGD scaffolds (NS). Chemical KCl stimulation induced an immediate isotonic shortening of the collagen scaffold. When a plateau was reached determining the maximum amplitude of shortening length (max SL), isometric conditions were quickly imposed on the scaffold, causing its length to immediately return to Lo. The scaffold then developed an active isometric tension (AT) equal to total isometric tension (TT) minus RT. Maximum shortening velocity (Vmax, in Lo·s^−1^) was obtained at zero-load. The Hill hyperbolic tension-velocity relationship [29] was derived from the peak velocity (V) of 8 to 10 isotonic levels of afterload, plotted against the isotonic force level normalized per CSA (T). Load-clamp increments were successively imposed from the total isometric tension (TT) to zero-load [28]. The tension-velocity (T-V) relationship was fitted according to the Hill equation (T+a) (V+b) = (TT+a) b where −a and −b were the asymptotes of the hyperbola which were determined by multilinear regression. The G curvature of the T-V relationship is equal to TT/a = V_max_/b [29].

### 2.2. The A. Huxley Formalism

**Figure 1:**
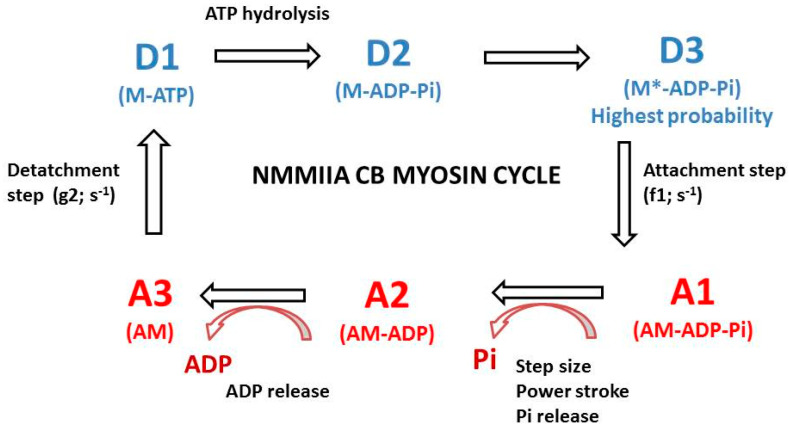
NMMIIA-CB cycle in myofibroblast. The cycle of the non-muscle NMMIIA is composed of six successive conformational steps. Three steps are detached (D1, D2, and D3) and three steps are attached (A1, A2, and A3). (1°) Transition A3 → D1. The ATP binding step induces myosin (M) detachment from actin (A) after ATP binding with the AM complex. The rate constant of CB detachment is g_2_. AM + ATP → A + M-ATP; (2°) Transition D1 → D2: ATP hydrolysis M + ATP → M-ADP-Pi. (3°) Transition D2 → D3 corresponds to M-ADP-Pi → M*-ADP-Pi; (4°) Transition D3 → A1 corresponds to the attachment state: M*-ADP-Pi binds with A and the attachment rate constant is f_1_: M*-ADP-Pi + A → AM-ADP-Pi; (5°) Transition A1 → A2 corresponds to the power stroke that is triggered by the release of Pi: AM-ADP-Pi → AM-ADP + Pi. The power stroke generates a single CB force and a myosin.

Huxley’s equations [17] are a powerful tool to determine the molecular properties of the NMMIIA molecular motor CBs and the probabilities of the different steps during the CB cycle (Figure 1). As a preliminary requirement, the use of this formalism needs to verify that the contractile scaffolds under study present a hyperbolic T-V relationship. Thus, values for the asymptotes -a and -b of the T-V relationship are introduced into the Huxley equations. The rate of total energy release (E_Hux_) and isotonic tension (P_Hux_) as a function of scaffold velocity (V) were obtained by the following classic equations [17,28]:

E_Hux_ = (N e) (h/2 ℓ) (f_1_/(f_1_ + g_1_)) {g_1_ + f_1_ (V/ϕ) [(1 − exp (−ϕ/V)]} (1)

P_Hux_ = N (w/ℓ) (f_1_/(f_1_ +g_1_)) {1 − (V/ϕ) [ (1 − exp (−ϕ/V)) (1 + (1/2) ((f_1_ + g_1_)/g_2_)^2^ (V/ϕ]} (2)

where w is the maximum mechanical work generated by a unitary CB (w/e = 0.75) and e is the free energy required to split one ATP molecule. Only one ATP is split per CB cycle. The standard free energy ΔG°′_ATP_ is about −60 kJ/mol, and e is equal to 10^−19^ J [30]. The tilt of the myosin head relative to actin varies from 0 to h; f_1_ is the maximum value of the rate constant for CB attachment; g_1_ and g_2_ are the maximum values of the rate constants for CB detachment; f_1_ and g_1_ correspond to a tilt of the myosin head from 0 to h; g_2_ corresponds to a tilt > h; ϕ is equal to (f_1_ + g_1_) h/2 = b; N is the number of cycling CB per mm^2^ of CSA at maximum isometric tension. The molecular step size h corresponds to the distance of translocation of the actin filament after the swing of the myosin head. The constant ℓ is the distance between two successive actin sites with which a myosin head could bind. In agreement with the A. Huxley conditions (ℓ >> h), h and ℓ values are h = 10 nm and ℓ = 28.6 nm respectively. The parameters f_1_, g_1_, and g_2_ were calculated using the following equations [31]:

G = f_1_/g_1_ (3)

g_1_ = 2wb/ehG (4)

g_2_ = 2V_max_/h (5)

kcat = (h/2 ℓ) × [(f_1_g_1_)/(f_1_ + g_1_)] (6)

po = (w/ℓ) × [(f_1_)/(f_1_ + g_1_)] (7)

Myosin content was calculated from the number of cycling myosin head per L of tissue and the Avogadro number. The maximum myosin ATPase activity is the product of myosin content and kcat which is the catalytic constant. 

### 2.3. Computation of CB Probabilities of the 6 States of the NMMIIA-CB Cycle 

The myosin CB cycle contains 6 main steps (Figure 1). There are three detached steps (D1, D2 and D3) and three attached steps (A1, A2 and A3). The method to determine the probabilities of the 6 states has been described in an earlier study [31]. Calculation of the probability of several states imposes the use of the A. Huxley formalism [17]. The probability of a given state is the ratio of the duration of this state and the overall duration of the CB cycle tc = 1/kcat. Molecular motors spend more time in detached state than in attached state [32,33]. Myosin CBs are “rower” molecular motors, as the sum of probabilities of detached states is more than 50%. This implies that 0.5 < (PD1+PD2+PD3) < 1 (where PD1 is the probability of step D1 and tD1 is the duration of this step. The most probable state was one of the 3 detached states. The probability PD1 was equal to tD1/tc = (1/g2)/tc = kcat/g2. Moreover, tD2 was approximated to 10 tD1 [34]. The probability PA1 was equal to tA1/tc = (1/f1)/tc = kcat/f1. During the power stroke and step size h, the energy was equal to eo = po * h. Probability PA2 was equal to eo/e (e: free energy required to split one ATP molecule). The most probable detached state was D3.

By convention, the lowest energy level (E_0_), coincided with the ground state and was equal to zero E_0_ = E_D3_ = 0 [15]. The lowest energy level was that of step D3. The ratio of probabilities of the most probable state and the least probable state was given by the relation: E_A3_ − E_D3_ = kT (ln P_D3_/P_A3_) = e = 10^−19^ J. As 0.5 < PD3 < 1 and E_A3_ − E_D3_ = kT ln (P_D3_/P_A3_), this implied that P_A3_ was ≪ to PA1, PA2, PD1 and PD2. Consequently, the least probable state was A3. Thus, the highest state level was E_A3_ = 10^−19^ J. Moreover, PA3+PD3 = 1 − (PA1+PA2+PD1+PD2). Since we know the ratio PD3/PA3 and the sum PA3 + PD3, we can therefore deduce PA3 and PD3.

### 2.4. Statistical Mechanics 

Living contractile tissues can exchange matter with the surroundings and produce ATP that drives the mechanical processes. One ATP is consumed per CB cycle, due to the one-to-one chemo-mechanical coupling [17]. The number of independent and distinguishable cycling CBs was equal to the number of ATP molecules that were consumed during contractile processes. The grand canonical ensemble represents a general and useful method if one wants to apply the statistical mechanics for studying complex open systems [15] such as collagen scaffolds seeded or not seeded with MSCs. Let **S** be an open contractile system in a container (**C**). Myosin CB, actin, and small soluble molecules (i.e., ATP, ADP and inorganic phosphate Pi) can be exchanged between **S** and **C**. Thus, the number of cycling CBs may fluctuate slightly with the number of non-cycling CBs, the latter becoming cycling CBs and vice versa. The system **S** was composed of all the active cycling myosin CBs individually found in one of six different states. Myosin CB were either attached to or detached from actin, and bound or not bound with ATP, ADP or Pi. The container **C** was composed of all the non-cycling myosin CBs, all the non-cycling actin molecules, and all the ATP, ADP and Pi that were not attached to the cycling myosin CBs. In the grand canonical ensemble, the average number of independent, non-interacting cycling myosin heads within **S** was determined from A. Huxley’s equations [17].

**Figure 2:**
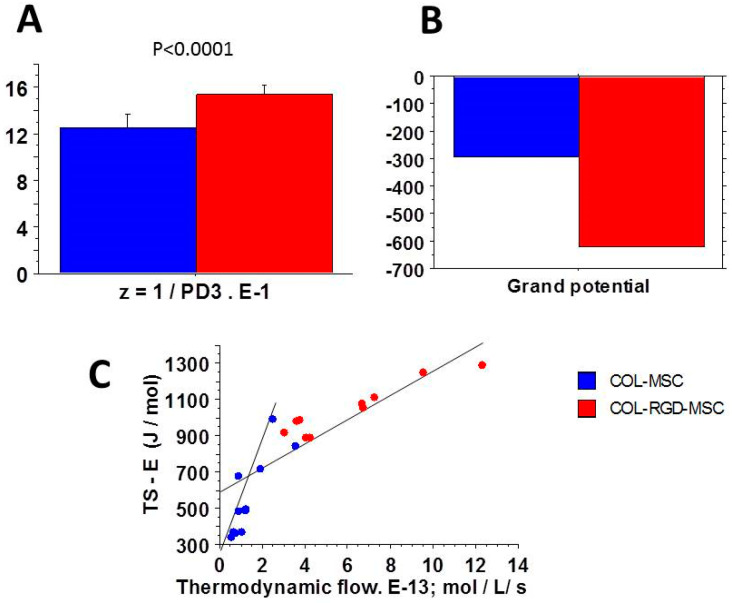
Parameters of statistical mechanics. (**A**): the microcanonical partition function (z) was significantly greater in COL-RGD-MSC scaffolds (red) than in COL-MSC scaffolds (blue); (**B**): the grand potential was significantly greater in COL-MSC-RDG scaffolds than in COL-MSC scaffolds; (**C**): there was a linear relationship between (TS-E) and the thermodynamic flow. When the thermodynamic flow tends towards zero, the chemical affinity tends towards zero and (TS-E) represents the value of the grand potential; y = 295 + 200 x, r = 0.84 in COL-MSC scaffolds; y = 620 + 45 x, r = 0.95 in COL-MSC-RGD scaffolds.

Let A be the chemical affinity of the CB cycle, S the statistical entropy, E the internal energy, and T (Kelvin) the temperature of S. The grand potential (Ψ) (Figure 2) is linked to E, S, A and T in agreement with the classic relationship Ψ = E − TS + A. Statistical entropy S is given by the equation S = −R∑_r_P_r_lnP_r_.

S is related to the dispersal of energy and measured the degree of disorder in a given system. In statistical mechanics, the state function internal energy (E) is the ensemble average of the sum of the microscopic kinetics (translations, rotations, and vibrations) and potential energy of the system.

The microcanonical partition function (z) was:

z = 1/Pmax = 1/PD3 (Figure 2A)(8)

We also have the classic thermodynamic equation: E − T S = −RT ln z:

E − T S = Ψ − A = −RT ln z = RT ln PD3(9)

Thus E = RT ln P_max_ + TS. The CB cycle stopped when the thermodynamic flow v (v = k_cat_ × mole number per liter) in **S** tended towards zero. In these conditions, the affinity A also tended towards zero. Consequently, E − TS tended towards Ψ (Figure 2B).

The extrapolation at thermodynamic flow equal to 0 of the E − TS versus the thermodynamic flow relationship was the ordinate of this relationship and was equal to Ψ (Figure 2C). Thus, the affinity A was calculated by the following equation:

A = Ψ − RT ln PD3(10)

The thermodynamic force was equal to A/T. When the affinity A is ≪ RT (R: gas constant; T: Kelvin temperature, i.e., RT ≈ 2500 J/mol), a chemical system operates near-equilibrium. A near-equilibrium, a chemical system evolves towards a stationary state when the thermodynamic force (A/T) varies linearly with the thermodynamic flow [22,35]. The change in entropy dS is the sum of two parts: dS = d_e_S + d_i_S, (d_i_S  ≥  0), in which d_e_S is the entropy change due to exchange of matter and energy with the exterior of the system and d_i_S is the entropy change due to irreversible processes within the system. 

In linear stationary systems, the entropy production rate (d_i_S/dt) is the product of the thermodynamic force (A/T) and the thermodynamic flow, i.e., d_i_S/dt = (A/T) × thermodynamic flow [35,36]; diS/dt can reach a minimum level that represents the criterion of stability of a stationary state. All the irreversible chemical processes are quantified by diS/dt. The higher the diS/dt, the further the chemical system moves far away from equilibrium. 

### 2.5. Statistical Analysis

Data were expressed as means ± standard deviation (SD). For comparisons between control scaffolds (*n* = 14) and RDG-scaffolds (*n* = 10), the Student’s unpaired t-test was used. When the p value was less than 0.05, the two groups were considered statistically different. We used the least squares method for linear regression. The asymptotes of Hill’s hyperbola were determined by the multilinear regression method.

## 3. Results

### 3.1. Near-Equilibrium Collagen Scaffolds Operated in a Linear Stationary Regime

A previous study from our laboratory showed that the MSCs introduced into both COL-MSC collagen scaffolds and COL-MSC-RDG collagen scaffolds massively differentiated into myofibroblasts that contained a very large number of NMMIIA molecular motors, responsible for contractile properties [28]. 

**Figure 3:**
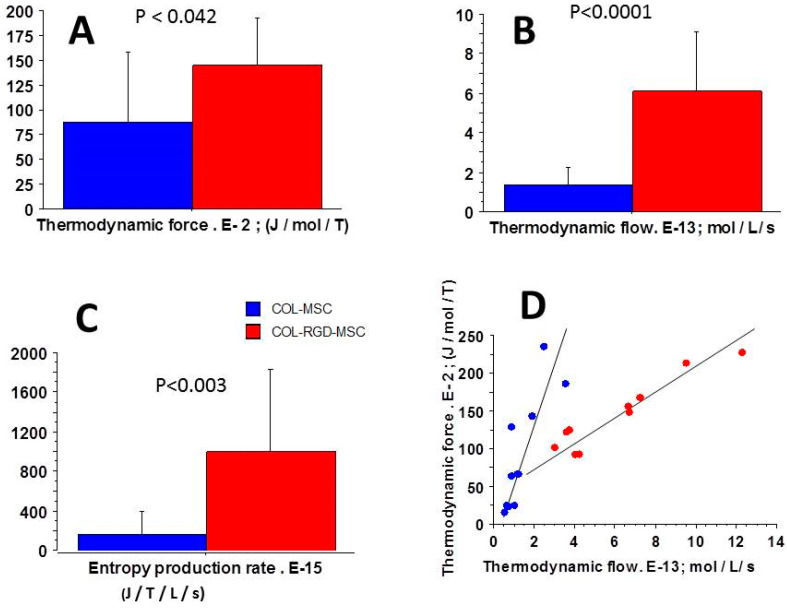
Parameters of statistical mechanics. (**A**): Thermodynamic force; (**B**): Thermodynamic flow; (**C**): Entropy production rate. These three parameters were significantly greater in COL-MSC-RDG scaffolds (red) than in COL-MSC scaffolds (blue); (**D**): There was a linear relationship between the thermodynamic force and the thermodynamic flow showing that both the 2 types of contractile scaffolds behaved in a stationary mode; y = −5 + 68 x; r = 0.84 in COL-MSC scaffolds; y = 48 + 15 x in COL-MSC scaffolds.

These open systems exchanging energy and matter with the outside made it possible to use the grand canonical ensemble for the determination of thermodynamic quantities. In myofibroblasts, the considerable number of actin-myosin crossbridges (CBs) per volume unit (Table 1) justified the use of statistical mechanics (SM). The chemical affinity was much less than 2500 J/mol in the two types of collagen scaffolds. It was necessary to show that these two systems operated near-equilibrium. In addition, the thermodynamic force (Figure 3A) and the thermodynamic flow (Figure 3B) varied linearly (Figure 3D), which showed that these two systems operated in a stationary regime.

### 3.2. Mechanical Parameters of COL-MSC and COL-MSC-RGD Scaffolds 

Three classic mechanical parameters of scaffolds were significantly greater in COL-MSC-RGD scaffolds than in COL-MSC scaffolds, i.e., total tension (TT), maximum velocity of shortening at zero-load (Vmax) and percentage of maximum shortening length (max SL) (Table 1). This indicates a positive inotropic effect induced by the tripeptide RGD. The number of CB per L of tissue (Table 1) was significantly greater in COL-MSC-RGD scaffolds than in COL-MSC scaffolds. However, the elementary CB force did not differ in the two types of scaffolds (Table 1).

### 3.3. Probabilities of the Six Conformational Steps of the NMMIIA CB Cycle

The probabilities of the 6 steps of the NMMIIA, i.e., PD1, PD2, PD3, PA1, PA2 and PA3 did not differ in the two types of collagen scaffolds (Table 2). Importantly, PD3 was slightly but not significantly decreased in COL-MSC-RGD scaffolds compared with COL-MSC scaffolds.

### 3.4. Molecular NMMIIA-CB Parameters of COL-MSC and COL-MSC-RGD Scaffolds

**Figure 4:**
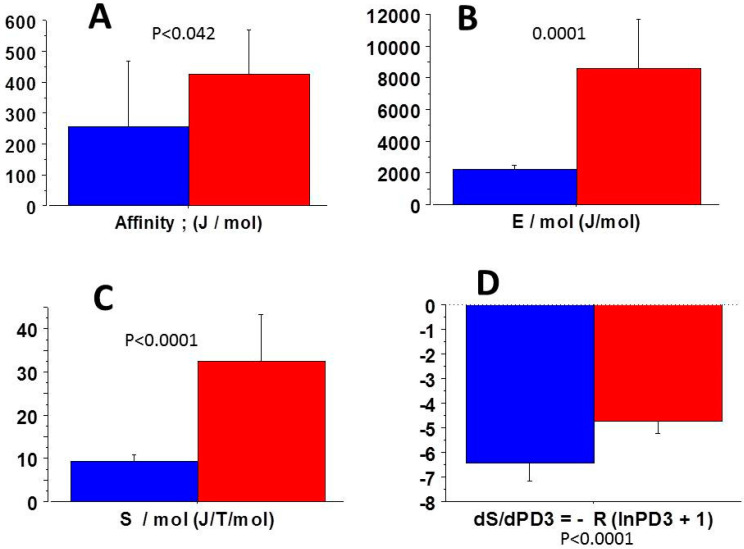
Parameters of statistical mechanics. (**A**): Chemical affinity; (**B**): Internal energy; (**C**): statistical entropy. These three parameters were significantly greater in COL-MSC-RDG scaffolds (red) than in COL-MSC scaffolds (blue); (**D**): Statistical entropy derivative according to probability PD3 (dS/dPD3) was significantly smaller in COL-MSC-RDG scaffolds than in COL-MSC scaffolds.

Chemical affinity (Figure 4A), statistical entropy (Figure 4C), internal energy (Figure 4B), micro canonical partition function (z) (Figure 2A), thermodynamic force (Figure 3A), thermodynamic flow (Figure 3B), and the entropy production rate (Figure 3C) were significantly greater in COL-MSC-RGD scaffolds than in COL-MEM scaffolds. The grand potential was significantly more negative in COL-MSC-RGD scaffolds than in COL-MSC scaffolds (Figure 2B).

### 3.5. Relationships Between Thermodynamic Quantities

**Figure 5:**
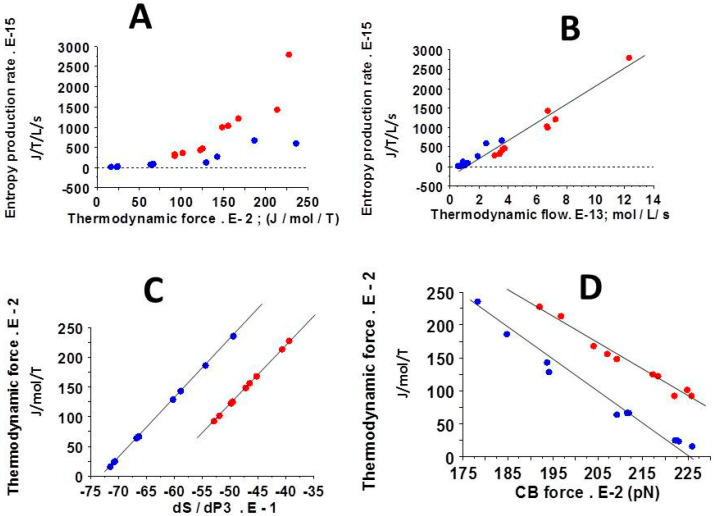
Parameters of statistical mechanics. (**A**): Non-linear relationship between the entropy production rate and the thermodynamic force. Influence of the thermodynamic force was moderately greater in COL-MSC-RDG (red) scaffolds than in COL-MSC scaffolds (blue); (**B**): Linear relationship between the entropy production rate and the thermodynamic flow; y = −212 + 216 * x; r = 0.97. Influence of the thermodynamic flow was significantly greater in COL-MSC-RDG scaffolds than in COL-MSC scaffolds; (**C**): Linear relationships between the thermodynamic force and the statistical entropy derivative according to probability PD3 (dS/dPD3); y = 7.3 + x; r = 1 in COL-MSC scaffolds; y = 6.2 + x; r = 1 in COL-MSC-RDG scaffolds. (**D**): Linear relationship between the thermodynamic force and the CB force: y = 990 − 4.4 x; r = 0.99 in COL-MSC scaffolds and y = 900 − 3.7 x; r = 0.87 in COL-MSC-RDG scaffolds.

The entropy production rate is the product of thermodynamic force and thermodynamic flow. Figure 5A shows that, for a given level of thermodynamic force, the presence of RGD increased the entropy production rate compared to scaffolds devoid of RGD. Moreover, the entropy production rate varied linearly with thermodynamic flow (Figure 5B). Figure 5C shows the perfect linear relationship between the thermodynamic force and dS/dPD3, which is inherent to the formalism of SM. Finally, the relationship between the thermodynamic force and the NMMIIA CB force varied linearly, with a slope moderately diminished in the presence of RGD. 

## 4. Discussion

### 4.1. Applying Statistical Mechanics (SM) to Collagen Scaffolds

SM together with the A. Huxley formalism allowed determining the microcanonical partition function (z) of the system [17]. The other macroscopic thermodynamic parameters of the system such as the statistical entropy, internal energy, grand potential, thermodynamic force, and the entropy production rate are byproducts of z and can be determined as well. All the thermodynamic information about a given system is contained in z which is related to the first and second laws of thermodynamics. It provides a clue of the average number of states that are thermally accessible at the temperature of the system under study [15]; z also reflects how molecular motors are partitioned over the available states. z was significantly increased in COL-MSC-RGD compared to COL-MSC scaffolds (Figure 2A) and was a hyperbolic function of PD3, which did not differ between the two types of scaffolds. The significant increase of the microcanonical partition function induced by the RGD accounted for the dramatic changes of the other thermodynamic quantities, i.e., E, S, thermodynamic force and entropy production rate (Figure 3 and Figure 4). 

### 4.2. Near-Equilibrium Thermodynamics

Living systems never stay at equilibrium and either stand near equilibrium or far away from equilibrium [35]. Living systems can be maintained near equilibrium through a flow of energy and matter. This requires a chemical affinity ≪2500 kJ/mol. This was observed for scaffolds functionalized or not with the RGD tripeptide (Figure 4A). The chemical affinity was significantly higher in RGD-collagen scaffolds than in collagen scaffolds. This means that the former behaved farther from equilibrium than the latter. Numerous enzymatic systems such as cytosolic phosphorylation potential and NAD+-NADH ratio in human erythrocytes [30,37], the Gibbs-Donnan near-equilibrium system in heart muscle [38] and myosin II in skeletal and heart muscles [16] have been shown similar near equilibrium behavior.

### 4.3. The Linear Stationary Regime

The second major thermodynamic property of MSCs-laden collagen scaffolds was the linear relationship between the thermodynamic force and flow (Figure 3A,B,D) indicating that the two types of collagen scaffolds operated in a linear stationary state. Under linear regime where the phenomenological laws of Onsager are valid [22], a near-equilibrium system can evolve to a stationary state [21]. Various biological systems behave in a linear stationary non-equilibrium state with exchanges of energy and matter with the exterior. This thermodynamic behavior has been previously observed in both heart and skeletal muscles and in the non-muscle contractile human placenta [16,31,39,40] as well as in biological non muscle systems [23,25,26,41]. 

### 4.4. Entropy Production Rate 

In stationary linear regimes where the thermodynamic force varies linearly with thermodynamic flow, Prigogine’s theorem can be applied [35]. In such linear stationary systems subject to flow of energy and matter, the total entropy production rate reaches a minimum value. Entropy production rate (diS/dt) quantifies irreversibility and is linked to irreversible chemical processes generated during the non-muscle NMMIIA CB cycle [35]. In RGD-collagen scaffolds, both thermodynamic force (A/T) and to a greater extent, thermodynamic flow contributed to the significant increase in entropy production rate (Figure 3A,B,D). The greater entropy production rate in RGD-collagen scaffolds indicated that they generated more irreversible biochemical processes and behaved farther from equilibrium than collagen scaffolds devoid of RGD. The precise molecular mechanisms explaining thermodynamic changes induced by RGD remain unknown. Some structural, biochemical or mechanical factors may be involved such as changes in the three-dimensional structure of the myosin head [42,43,44,45], changes in structure or function of the regulatory and essential myosin light chains, changes in content or activity of the myosin light chain kinase and in the calcium-calmodulin complex [46,47]. It should be mentioned that a simple switch of the electrical stimulation from twitch mode to tetanus mode induces changes in the rate of entropy production [40]. Covalent binding of the RGD of fibronectin to the collagen in the extracellular matrix (ECM) improved in vitro the thermodynamic properties of myofibroblasts (Figure 2, Figure 3 and Figure 4). Myofibroblasts recognize the RGD during fibrillogenesis. The linear RGD peptide that is present on fibronectin is essential for the interaction with integrin receptors on myofibroblasts. Integrins are heterodimers of alpha and beta subunits that bind to ECM on the outside of the cell [48]. Integrin activation favors cell adhesion and the connection of ECM to the actin cytoskeleton. On the inside of the cell, the integrin cytoplasmic tails recruit various actin binding proteins, such as talin and vinculin present in myofibroblasts. In response to mechanical force, the integrin tails undergo conformational changes. These changes promote talin binding which in turn stimulates integrins to adopt an active conformation in association with ECM. Talin links activate integrins to actin via direct binding or through vinculin promoting focal adhesion formation and traction force [49]. In our study, the increase in entropy production rate observed in RGD-scaffolds was mainly due to the drastic increase in thermodynamic flow (Figure 3B), as a consequence of the increase in molar concentration of non-muscle NMMIIA CB myosin (Table 1).

### 4.5. Probabilities of the Steps of the NMMIIA CB Cycle

The presence of the RGD motif induced no significant changes in the probabilities of each step of the NMMIA CB cycle. PD3 probability is prominent for statistical mechanics because the highest probability of a given system is inversely proportional to the microcanonical partition function (z) (see Materials and Methods; z = 1/PD3). There was only a small and non-significant decrease of PD3 in RGD-collagen scaffolds compared to collagen scaffolds. However, due to the hyperbolic relationship between z and PD3, z increased significantly in RGD scaffolds. This explained why all the thermodynamic parameters depending on z namely, statistical entropy, internal energy, thermodynamic force, chemical affinity and the entropy production rate were significantly different between the two scaffold types. 

### 4.6. Thermodynamic Specificity of the Non-Muscle Myosin NMMIIA

The non-muscle myosin NMMIIA [3,11,12] is an essential component of myofibroblasts [1] in the human placenta [3,12]. Human placenta is a non-muscle contractile tissue [4,5,6]. The molecular kinetics of NMMIIA [13] has been found to be particularly slow compared to those of the muscle myosin MMI and II [16]. The molecular motor present in collagen scaffolds laden with MSCs was the NMMIIA [28], and its molecular characteristics were similar to those of the human placenta [14]. Moreover, in striated and smooth muscles, statistical entropy, chemical affinity, and thermodynamic force were roughly of the same order of magnitude, excepted for non-muscle contractile structures (collagen scaffold and human placenta) where thermodynamic flow and production entropy rate were particularly low and for heart muscle where thermodynamic flow and production entropy rate were particularly elevated [16]. 

## 5. Conclusions

Human MSCs seeded in collagen scaffolds used in this study represent an engineered tissue with contractile properties that were close to those of the human placenta. Both systems contain the non-muscle myosin NMMIIA molecular motor whose molecular kinetics are extremely slow. Collagen scaffolds seeded with MSCs with or without RGD operated near equilibrium, as evidenced by the chemical affinity ≪ 2500 J/mol. They remained in a stationary state as evidenced by the linearity between the thermodynamic force and flow. The functionalization of the collagen scaffolds by covalent fixation with the linear tripeptide Arg-Gly-Asp (RGD) of fibronectin induced a significant positive inotropic effect and modified the thermodynamic parameters such as statistical entropy, internal energy, chemical affinity thermodynamic force, thermodynamic flow, and the rate of entropy production, which led to an increase in irreversible processes. The functionalization of the collagen scaffolds by covalent fixation with the linear tripeptide Arg-Gly-Asp (RGD) could be useful, in particular in cardiac or cutaneous pathologies, where the inner tissue tension is particularly high. Adding RGD to the collagen scaffold could strengthen the scar function and represent a clinical advantage.

## Figures and Tables

**Table 1:** Classical mechanical parameters of COL-MSC and COL-MSC-RGD scaffolds and molecular properties of NMMIIA CBs.

	COL-MSC *n* = 14	COL-MSC-RGD *n* = 10	*p*
Tension (TT) (mN/mm^2^)	0.266 ± 0.103	0.642 ± 0.212	0.0001
max SL (L/Lo)	0.012 ± 0.008	0.020 ± 0.014	0.020
Vmax (Lo/s)	0.002 ± 0.001	0.004 ± 0.001	0.006
Eff. max (%)	38 ± 3	38 ± 3	0.857; NS
CB force (po) (pN)	2.1 ± 0.2	2.1 ± 0.1	0.865; NS
CB mole/L (E-11)	4.23 ± 1.55	10.27 ± 30	0.0001
CB number/L (E13)	2.55 ± 0.90	6.18 ± 1.99	0.0001

**Table 2:** Probabilities of the 6 steps of the NMMIIA CB cycle.

	COL-MSC *n* = 14	COL-MSC-RGD *n* = 10	*p*
PD1	0.008 ± 0.006	0.009 ± 0.005	0.618; NS
PD2	0.082 ± 0.056	0.094 ± 0.053	0.618; NS
PD3	0.825 ± 0.068	0.813 ± 0.062	0.658; NS
PA1	0.037 ± 0.011	0.036 ± 0.010	0.865; NS
PA2	0.041 ± 0.003	0.042 ± 0.003	0.865; NS
PA3. E-11	1.794 ± 0.147	1.766 ± 0.134	0.653; NS
